# Supplementary material for: A workflow for automatic, high precision livestock diagnostic screening of locomotor kinematics
Source: Front Vet Sci. 2023 Mar 7;10:1111140. doi: 10.3389/fvets.2023.1111140 (PMC10028250; doi:10.3389/fvets.2023.1111140)
Supplement: Supplementary file 1 [file Table_1.pdf]

Supplementary Material

1 DETAILED MODELING RESULTS

Detailed modeling results are presented in Table S1.

Table S1. Asterisk (*) indicates slopes for which the credible interval did not include zero. FL: forelimb, HL: hindlimb, dyn.p.: dynamic posture, coord.: coordination, diml.: dimensionless, d.s.: dimensionless stride, eROM: effective range of motion.

	category	parameter	age (h)	age (log)	size PC1	mass (kg)
0	model	intercept	+4.86	+1.68	−3.25	+1.38
1	subject	female → male	+0.05	+0.00	−0.38 *	−0.04
2	gait	log. FL clearance	−0.10	−0.08	+0.09	−0.02
3	gait	log. HL clearance	+0.94 *	+0.32 *	−0.65 *	−0.15 *
4	gait	FL duty factor	+1.11	+0.37	+0.18	+0.07
5	gait	HL duty factor	+0.67	+0.24	+0.85 *	+0.11
6	gait	d.s. distance	+1.32	+0.31	+0.45	+0.18
7	gait	d.s. frequency	+3.12	+1.43	+1.62	+0.15
8	gait	diml. speed	−1.10	−0.64	+0.82	+0.00
9	gait	hindlimb phase	−2.04	−0.56	−1.98	+0.05
10	gait	head angle	+0.26	+0.14	+1.44 *	+0.18
11	dyn.p.	mean hip angle	+2.55 *	+0.59 *	−1.07 *	−0.45 *
12	dyn.p.	hip eROM	+2.64	+0.94	−1.34	−0.52 *
13	dyn.p.	mean stifle angle	+0.67	+0.10	−2.01 *	−0.12
14	dyn.p.	stifle eROM	−1.85	−0.22	−1.66 *	−0.13
15	dyn.p.	mean tarsal angle	−1.18	−0.59	+0.44	+0.23 *
16	dyn.p.	tarsal eROM	−1.92	−0.88 *	+2.98 *	+0.60 *
17	dyn.p.	mean shoulder angle	+1.06	+0.07	+0.47	+0.06
18	dyn.p.	shoulder eROM	−0.34	−0.17	−0.65	+0.01
19	dyn.p.	mean elbow angle	−1.00	−0.55	+2.79 *	+0.20
20	dyn.p.	elbow eROM	+3.22	+0.96	+0.11	−0.30
21	dyn.p.	mean carpal angle	−2.08 *	−0.98 *	+1.69 *	+0.04
22	dyn.p.	carpal eROM	−0.24	+0.21	−0.84	+0.16
23	coord.	CC1	+0.60	+0.31 *	+0.10	−0.05
24	coord.	CC2	+0.50	+0.15	−0.24	−0.08
25	coord.	CC3	−0.25	+0.00	+0.88 *	+0.07
26	coord.	CC4	−0.62	−0.26	+0.13	+0.04
27	coord.	CC5	−0.84	−0.25	−0.16	+0.04
28	coord.	CC6	−1.49	−0.51 *	−0.10	+0.07
29	coord.	CC7	+0.24	+0.22	−0.76	−0.08
30	coord.	CC8	−1.19	−0.24	−0.51	+0.02
31	coord.	CC9	−2.96 *	−0.83 *	+0.66	+0.06
32	coord.	CC10	−0.22	+0.06	−0.60	+0.02
33	coord.	CC11	−2.05 *	−0.67 *	+0.10	+0.08
34	coord.	CC12	−0.03	−0.10	+0.35	−0.01
35	model	ϵ	±1.81	±0.56	±0.98	±0.20
